# Association of human papillomavirus on risk of HIV acquisition in African women: analyses from MTN-020/ASPIRE

**DOI:** 10.1093/jnci/djaf336

**Published:** 2025-11-19

**Authors:** Christine L Hathaway, Elizabeth R Brown, Stephen Cherne, Alyssa L Sepulveda, Zvavahera M Chirenje, Nitesha Jeenarain, Logashvari Naidoo, Samantha Siva, Nishanta Singh, Kubashni Woeber, Zakir Gaffoor, Brenda Gati Mirembe, Flavia Matovu Kiweewa, Leila E Mansoor, Lameck Chinula, Sufia Dadabhai, Nelly R Mugo, Thesla Palanee-Phillips, Ruanne V Barnabas

**Affiliations:** Division of Infectious Diseases, Massachusetts General Hospital, Boston, MA, United States; University of Washington School of Medicine, Seattle, WA, United States; Vaccine and Infectious Disease Division, Fred Hutchinson Cancer Center, Seattle, WA, United States; Department of Biostatistics, University of Washington, Seattle, WA, United States; Department of Laboratory Medicine and Pathology, Harborview Medical Center, Seattle, WA, United States; University of Texas Rio Grande Valley School of Medicine, Edinburg, TX, United States; Department of Obstetrics/Gynecology and Reproductive Science, University of California San Francisco, San Francisco, CA, United States; University of Zimbabwe-Clinical Research Center, Harare, Zimbabwe; South African Medical Research Council, HIV and Other Infectious Diseases Research Unit, Durban, South Africa; South African Medical Research Council, HIV and Other Infectious Diseases Research Unit, Durban, South Africa; South African Medical Research Council, HIV and Other Infectious Diseases Research Unit, Durban, South Africa; South African Medical Research Council, HIV and Other Infectious Diseases Research Unit, Durban, South Africa; South African Medical Research Council, HIV and Other Infectious Diseases Research Unit, Durban, South Africa; South African Medical Research Council, HIV and Other Infectious Diseases Research Unit, Durban, South Africa; Makerere University—Johns Hopkins University Research Collaboration, Kampala, Uganda; Makerere University—Johns Hopkins University Research Collaboration, Kampala, Uganda; Centre for the AIDS Programme of Research in South Africa, University of KwaZulu-Natal, Durban, South Africa; University of North Carolina Project Malawi, Lilongwe, Malawi; Department of Obstetrics and Gynecology, University of North Carolina, Chapel Hill, NC, USA; Johns Hopkins Research Project-Kamuzu University of Health Sciences, Blantyre, Malawi; Johns Hopkins School of Public Health, Baltimore, MD, United States; Department of Global Health, University of Washington, Seattle, WA, United States; Kenya Medical Research Institute, Nairobi, Kenya; Wits RHI, Faculty of Health Sciences, University of the Witwatersrand, School of Public Health, Johannesburg, South Africa; Department of Epidemiology, School of Public Health, University of Washington, Seattle, WA, United States; Division of Infectious Diseases, Massachusetts General Hospital, Boston, MA, United States; Harvard Medical School, Boston, MA, United States

## Abstract

**Background:**

Observational data on the synergy between human papillomavirus (HPV) infection and risk for HIV acquisition are needed. HPV clearance, associated with an influx of cells targeted by HIV, may increase HIV risk. This study examined the association between HPV and HIV acquisition using endocervical swabs collected in MTN-020/ASPIRE.

**Methods:**

Healthy, sexually active women without HIV participating in the ASPIRE dapivirine ring study in Malawi, South Africa, Uganda, and Zimbabwe provided endocervical swabs monthly, which were tested for HPV-DNA. HPV status was classified into prevalent, persistent (HPV+ for at least 4 months), HPV clearance (HPV+ followed by HPV−), HPV acquisition (HPV− followed by HPV+), and remaining HPV positive (2 subsequent HPV+). Cox time-varying regression models were used to assess associations between HPV states and HIV acquisition.

**Results:**

Among 91 HIV acquisition endpoints, HPV clearance increased HIV risk for high-risk types (16/18/31/33/35/45/52/58) (HR = 2.40, 95% CI = 1.59 to 3.62). Remaining HPV+ also showed a moderately increased risk (HR = 1.59, 95% CI = 1.07 to 2.35), while prevalent, persistent, and HPV acquisition events showed non-significant associations. Elevated HIV risk was also observed for clearance of HPV16/18, other high-risk types, low-risk types, and HPV6/11. There was also a dose-response relationship, with HIV risk increasing by 1.75-fold (95% CI = 1.55 to 1.96) for each additional HPV type cleared.

**Conclusions:**

HPV clearance-related immune activation is strongly linked to HIV acquisition, likely due to increased CD4+ T cells and inflammation. These findings support HPV vaccination as a potential HIV prevention strategy and highlight the need to integrate HIV prevention into cervical cancer programs.

## Introduction

Human papillomavirus (HPV) is one of the most common sexually transmitted viruses worldwide, with a lifetime acquisition risk of 80% for both men and women by age 45.^1^ Roughly 80%-90% of HPV infections clear spontaneously within 24 months, but infections that persist are more likely to lead to squamous intraepithelial lesions and have a greater risk of neoplastic transformation.^2^

Cervical cancer incidence is greatest in Eastern and Southern Africa, with incidence rates of 40 and 36 cases per 100 000 women-years, respectively.^3^ These regions also have a high prevalence of sexually transmitted HIV, and women living with HIV are 6 times more likely to acquire HPV and have a lower rate of clearing infections or precancerous lesions.^4^ However, the risk for HIV acquisition with HPV infection is not as clearly established. Previous studies suggest a 2- to 3-fold increase in HIV risk among women with a prevalent HPV infection.^5-8^ It is unclear whether this increased risk is driven by HPV prevalence, recent acquisition, persistence, or clearance. Some evidence points to HPV clearance as a key factor due to immune activation and the increased presence of HIV target cells, though past studies were limited by infrequent sampling.^8^ Data have been sparse, in part, due to absence of longitudinal, regularly collected cervical specimens with confirmation of outcomes such as HIV acquisition and HPV infection status.

We leveraged endocervical specimens collected monthly in MTN-020/ASPIRE, a randomized trial of the dapivirine vaginal ring for HIV prevention in Malawi, South Africa, Uganda, and Zimbabwe, to strengthen the evidence on the association of HPV infection with the risk of HIV acquisition.

## Methods

### Study participants

Our study utilized specimens from MTN-020/ASPIRE, a randomized efficacy and safety trial of a monthly vaginal ring containing dapivirine for HIV prevention, among women aged 18 to 45 years, in Malawi, South Africa, Uganda, and Zimbabwe. ASPIRE enrolled participants from 2012 to 2015 and was approved by the institutional review boards and ethics committees at participating institutions.^9^

Participants were randomized to receive either a silicone elastomer vaginal matrix ring containing 25 mg of dapivirine or a placebo vaginal ring. Monthly visits included HIV-1 testing using 2 rapid tests, with confirmatory Western blot and RNA PCR if either rapid test was positive. Sexually transmitted infection (STI) testing was conducted semi-annually or as clinically indicated and included trichomonas rapid testing, NAAT for gonorrhea and chlamydia, and syphilis serology. Endocervical swabs were collected monthly, cervical cytology smears were done at enrollment and the Product Use End Visit (PUEV), and behavioral data on condom use and sexual activity were collected quarterly.

**Figure 1.**
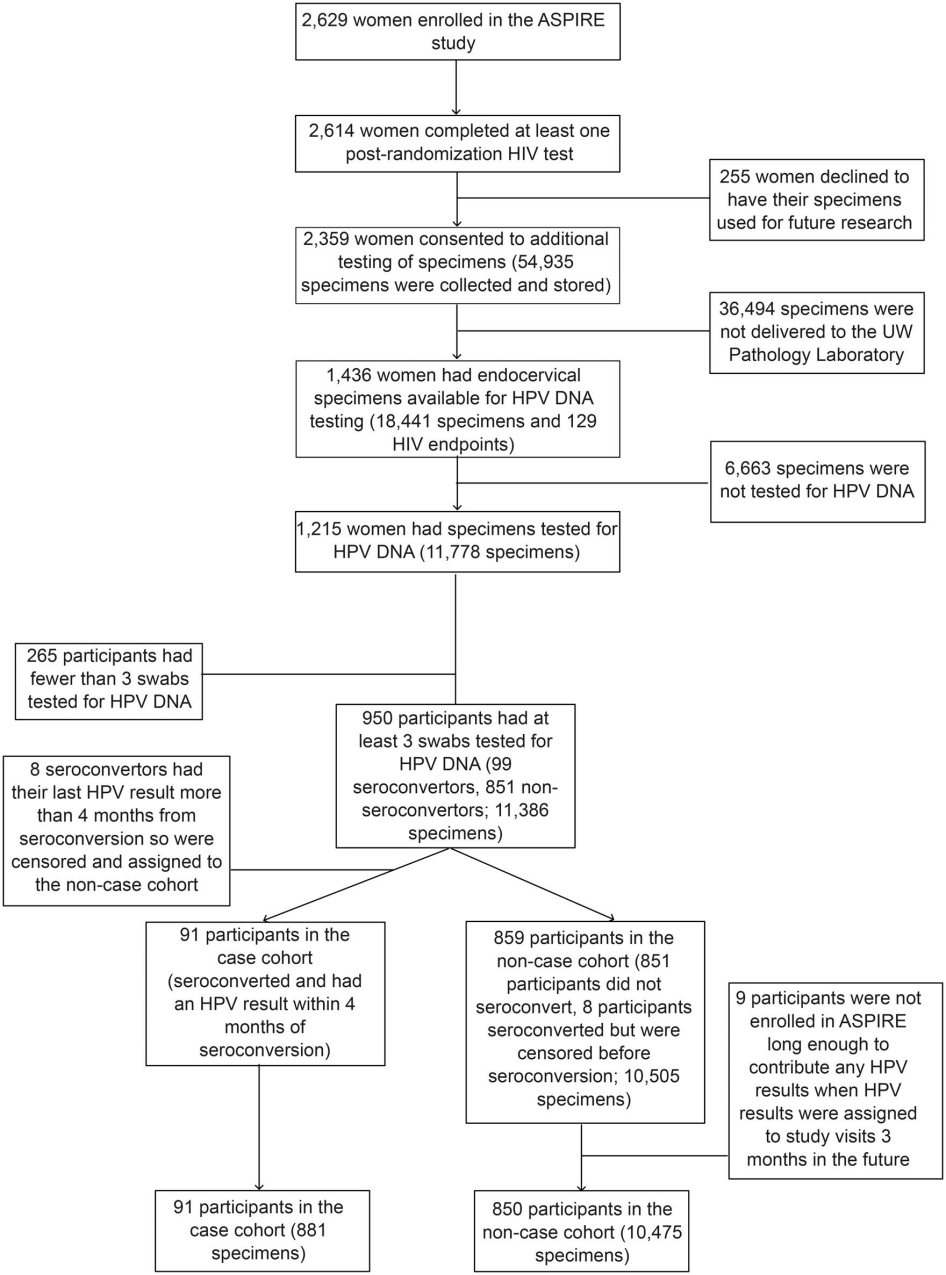
CONSORT diagram representing the inclusion of ASPIRE participants into the observational cohort. Inclusion criteria included completing at least 1 post-randomization HIV test, consenting to additional testing of specimens, and having at least 3 endocervical swabs tested for HPV DNA. Endpoints were defined as seroconversion with an HPV result within 4 months prior. Participants were censored if they did not seroconvert during ASPIRE or seroconverted but had their last HPV DNA result more than 4 months prior.

For this observational study, HPV-DNA testing was conducted on a convenience sample of endocervical swabs, oversampling participants who seroconverted, had abnormal cytology, or provided ≥12 swabs to optimize longitudinal analysis. For participants who acquired HIV, swabs were selected from visits up to 4 months before seroconversion. For those who did not acquire HIV, specimens were selected from enrollment through study exit. Sample selection details are shown in Figure 1. Additional details about ASPIRE specimens and HPV-DNA testing are described in Figures S2 and S3 and Tables S1-S3.

### HPV-DNA testing laboratory procedures

Dry swabs stored at −80°C were shipped to the University of Washington Pathology Laboratory at Harborview Medical Center. Swabs were suspended in 500 µL of PBS, 200 µL of which was withdrawn for DNA extraction. DNA extraction was performed on the Roche MagNa Pure 96 using the MagNa Pure 96 DNA and Viral NA Small Volume kit. HPV-DNA testing was performed using the Seegene Anyplex II HPV28 Detection kit, which can detect 28 HPV types (High Risk: 16/18/26/31/33/35/39/45/51/52/53/56/58/59/66/68/69/73/82, Low Risk: 6/11/40/42/43/44/54/61/70).^10^

### Cohort selection and statistical analysis

Selected participants were those with at least 3 swabs tested for HPV-DNA. The endpoint was HIV seroconversion. Participant follow-up was included until seroconversion or their final HPV-DNA result visit if they did not seroconvert during ASPIRE or seroconverted but their last HPV-DNA result was more than 4 months prior.

Baseline characteristics collected at the enrollment visit (age, education, country, study arm, marital status) and time-varying characteristics collected at the follow-up visits (condom use at the last sex encounter, multiple sex partners, and the presence of any STIs including syphilis, trichomoniasis, gonorrhea, and chlamydia) were compared between cases and non-cases using Chi-square tests for categorical variables and analysis of variance tests for continuous variables.

HPV types were classified into the 8 carcinogenic HPV types relevant for screening per the WHO (8 cHPV—HPV 16/18/31/33/35/45/52/58), bivalent vaccine (2vHPV—HPV 16/18), other carcinogenic HPV types (other cHPV—HPV 39/51/56/59/66/68/73), low-risk HPV types (lrHPV—HPV 26/40/42/53/54/61/69/70/82), non-carcinogenic HPV types (HPV 6/11), and any HPV type.^11^ High-risk and low-risk classifications followed the International Agency for Research on Cancer classification scheme.^11^^,^^12^ We classified HPV infections as prevalent (HPV+ at any 1 visit), persistent (HPV+ for the same type for at least 4 months, aligning with the lower bound interval definition of persistence used in prior clinical trials^13^), HPV clearance (HPV+ following HPV− at a subsequent swab for the same HPV type), HPV acquisition (HPV− following HPV+ at a subsequent swab for the same HPV type), and remaining HPV positive (HPV+ following HPV+ at a subsequent swab for the same HPV type). The HPV clearance, acquisition, and remaining HPV positive classifications align with those used in prior observational studies.^8^

Because the population in this analysis was not selected at random, inverse probability sampling weights were calculated to reflect the population of ASPIRE participants who consented to future specimen use. For those who seroconverted or had an abnormal cervical cytology result, weights were stratified by age group (18-21, 22-26, and 27+), study site, HIV status, and cytology result. For HIV-negative participants with normal cytology, weights were stratified by study site and age group.

Discrete-time survival models using time-varying covariate Cox regression estimated HIV seroconversion risk over time relative to HPV status assessed 3 months prior, with hazard ratios (HRs) and 95% confidence intervals (CIs). Missing HPV data at intermediate time points were imputed using last observation carried forward. The 3-month adjustment was made to ensure that HPV status reflected the time point closest to HIV acquisition, based on evidence that HPV infection detected approximately 3 months prior to seroconversion best represents the infection status at or near the time of seroconversion.^14^ In the Cox models, start time was defined as 3 months after a participant’s second HPV result, which was required to characterize events like immune activation and HPV persistence. Event was defined as the month of seroconversion. Participants were censored 3 months after their last available HPV result if they did not seroconvert or seroconverted more than 4 months from their last HPV-DNA result. Participants enrolled in ASPIRE for less than 5 months were excluded due to insufficient data. The hazard function at time (*t*) for an individual is:


$$h(t)=h_{S}(t)e\mathrm{xp}(\sum_{i=1}^{p}\beta X(t-3)+\alpha 'Z)$$

where *h_s_*(*t*) represents the baseline hazard function for each period (*s*), β represents the regression coefficient corresponding with the time-varying covariate, and *X*(*t-3*) represents the time-varying covariate (HPV status) measured 3 months earlier at time *t-3*. Cox models were stratified by study site (as indicated by the subscript S on the baseline hazard) and adjusted by age at enrollment (ages 18-21, 22-26, and 27+), secondary education, condom use at the last sex encounter, multiple sex partners, any STIs, and study arm (contained in the vector Z and linked to the baseline hazard through the coefficient vector α).

The primary analysis estimated HIV seroconversion risk based on infection by the 8 cHPV categorized as prevalent, persistent, HPV clearance, and HPV acquisition. Further analysis of HPV clearance was performed by grouping HPV types into any HPV, 2vHPV, other cHPV, lrHPV, and HPV6/11. We also evaluated the association of HIV risk with the number of concurrent HPV infections of any HPV type (categorical and continuous). Separate Cox models were used for each classification to assess their independent associations with HIV seroconversion. Because an individual can be infected with multiple HPV types simultaneously, a swab was classified under a category if any HPV type within the group met the criteria. Figure S1 illustrates an example of these classifications.

Additionally, supplementary analyses were conducted to explore the association of persistent HPV with abnormal cervical cytology results. Methods of this analysis are described in Section SIIIc. All analyses were performed in R (version 4.2.0).

## Results

### Characterizing HPV infections

A total of 11 778 specimens from 1215 ASPIRE participants were tested for HPV-DNA. This included 129 participants who seroconverted and 1086 participants who remained HIV-negative during ASPIRE.

Among the 1215 participants with HPV-DNA results, the prevalence of any HPV infection at the baseline HPV-DNA swab was 71% (859/1215). The prevalence of 2vHPV was 18%, 8 cHPV was 45%, other cHPV was 33%, lrHPV was 35%, and HPV 6/11 was 9%. More than half of the participants with a baseline HPV-DNA infection were infected with 2 or more HPV types (62.2%, 534/859). HPV results are described further in Tables S5 and S6.

### Association of HPV infection with risk of HIV seroconversion

Based on the inclusion criteria, 91 participants seroconverted while 850 participants were censored, as shown in Figure 1. Demographic differences were observed between groups (Table 1) based on mean age, study arm, and presence of any STIs. Cases were younger (mean age 25.0 vs 27.9, *P* < .001) and more likely to be in the placebo arm (63% vs. 49%, *P* = .015). Other baseline characteristics were similar, including education, country, and marital status. Time-varying factors like condom use (59% vs. 62%, *P* = 1.000) and multiple sex partners (14% vs. 9%, *P* = .157) were comparable at the enrollment visits. However, STI prevalence at enrollment was higher among cases (59% vs. 34%, *P* < .001). Characteristics of ASPIRE participants not included in the observational study are described in Table S4.

**Table 1.** Demographic characteristics and sexual behaviors of study participants at ASPIRE enrollment.

	Censored participants^a^ (*n* = 850)	Endpoint participants^b^ (*n* = 91)	Total (*n* = 941)	*P* ^c^
*Characteristics at enrollment*				
Mean age (SD)	27.9 (6.2)	25 (5)	27.6 (6.1)	<.001
Education completed				.141
No schooling completed	61 (7%)	7 (8%)	68 (7%)	
Primary school	384 (45%)	51 (56%)	435 (46%)	
Secondary school	353 (42%)	31 (34%)	384 (41%)	
College or university	52 (6%)	2 (2%)	54 (6%)	
Country				.644
Malawi	27 (3%)	4 (4%)	31 (3%)	
South Africa	627 (74%)	71 (78%)	698 (74%)	
Uganda	60 (7%)	5 (5%)	65 (7%)	
Zimbabwe	136 (16%)	11 (12%)	147 (16%)	
Study arm				.015
Dapivirine	437 (51%)	34 (37%)	471 (50%)	
Placebo	413 (49%)	57 (63%)	470 (50%)	
Married				.074
Yes	228 (27%)	16 (18%)	244 (26%)	
No	622 (73%)	75 (82%)	697 (74%)	
*Time varying characteristics at enrollment* ^d^				
Condom use during last sex encounter				1.000
Yes	524 (62%)	54 (59%)	578 (61%)	
No	326 (38%)	37 (41%)	363 (39%)	
Multiple sex partners				.157
Yes	77 (9%)	13 (14%)	90 (10%)	
No	773 (91%)	78 (86%)	851 (90%)	
Any STIs^e^				<.001
Yes	288 (34%)	54 (59%)	342 (36%)	
No	562 (66%)	37 (41%)	599 (64%)	

Abbreviations: SD = standard deviation; STI = sexually transmitted infection.

a Participants were censored in the time-varying Cox models if they did not seroconvert or seroconverted more than 4 months from their last HPV-DNA result.

b Endpoint participants were those who seroconverted within 4 months from their last HPV-DNA result.

c Estimated from analysis of variance tests for continuous variables and Chi-square tests for categorical variables.

d These characteristics are time-varying covariates in the Cox models, but for this table, are summarized at enrollment.

e Syphilis, trichomoniasis, gonorrhea, and chlamydia.

**Figure 2.**
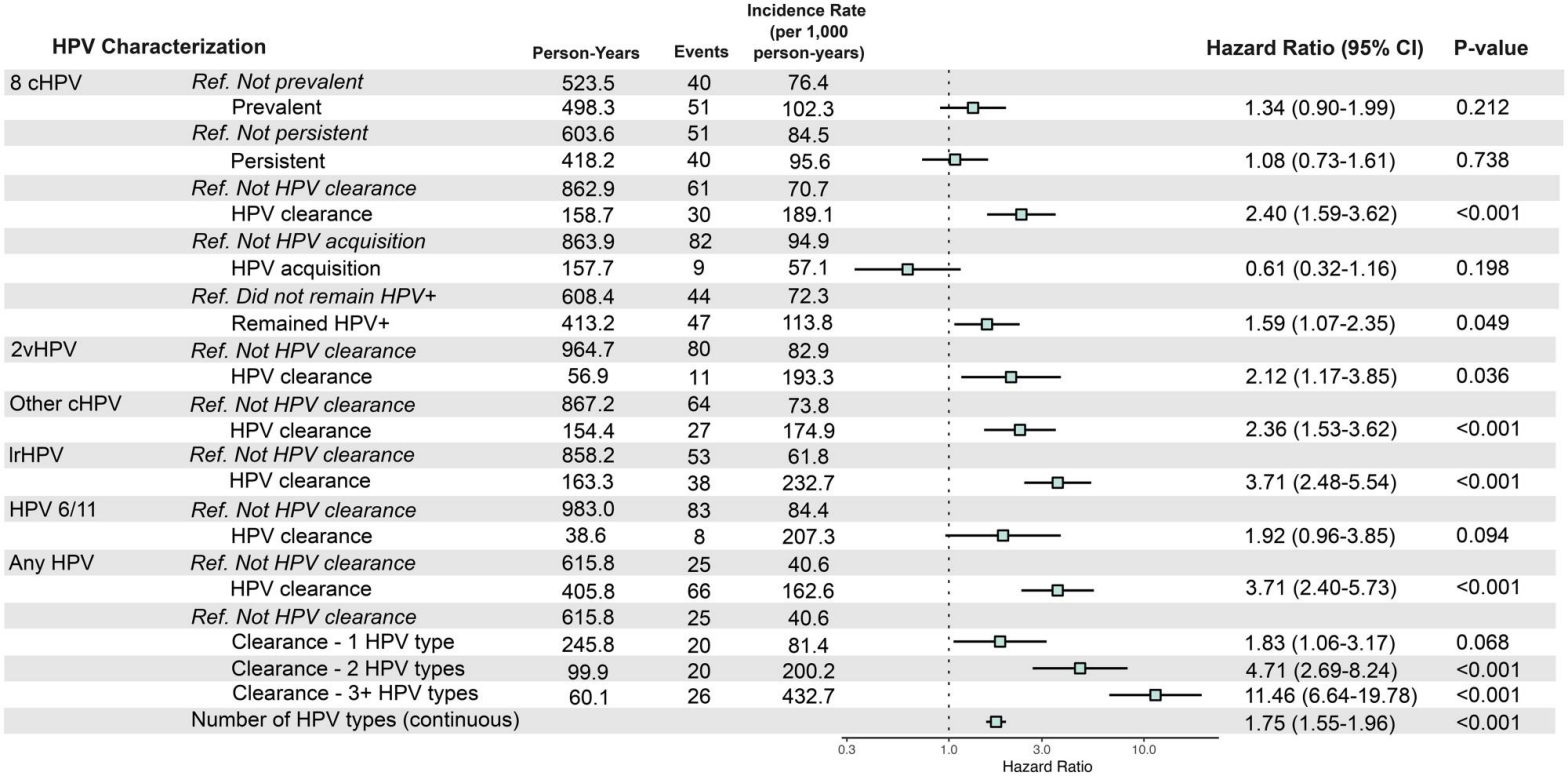
Adjusted hazard ratios from Cox time-varying models of HIV risk based on time-varying HPV status 3 months prior. Each time-varying HPV covariate value was analyzed separately in distinct Cox models. Models were adjusted by age group, study site, completion of secondary education, condom use at the last sex encounter, having multiple sex partners, the presence of any STIs, and ASPIRE study arm. Reference HPV characterization events indicate the absence of an HPV event at the corresponding time point. Abbreviations: 8 cHPV = 8 carcinogenic HPV types relevant for screening per the WHO (HPV16/18/31/33/35/45/52/58); 2vHPV = bivalent HPV vaccine types (HPV16/18); other cHPV = other carcinogenic HPV types (HPV39/51/56/59/66/68/73); lrHPV = low-risk HPV types (excluding HPV 6/11).

For the 8 cHPV, HPV clearance 3 months prior significantly increased HIV risk (HR = 2.40, 95% CI = 1.59 to 3.62; Figure 2). Remaining HPV+ also elevated HIV risk (HR = 1.59, 95% CI = 1.07 to 2.35). Prevalent, persistent, and HPV acquisition events showed minor, non-significant associations (HRs = 1.34, 1.08, and 0.61, respectively). Cox time-varying models showed similar HIV risk for 2vHPV HPV clearance (HR = 2.12, 95% CI = 1.17 to 3.85). Other cHPV and lrHPV HPV clearance were significantly associated with HIV acquisition (HR = 2.36, 95% CI = 1.53 to 3.62 and HR = 3.71, 95% CI = 2.48 to 5.54, respectively), as was combined HPV types (HR = 3.71, 95% CI = 2.40 to 5.73).

There was also a dose-response with the number of HPV types undergoing HPV clearance. For all HPV types, having 1 HPV type clearance 3 months prior was associated with a 1.83 (1.06 to 3.17) times greater risk of HIV acquisition compared to no HPV clearance event; the point-estimate for the hazard ratio doubled to 4.71 (2.69 to 8.24) for 2 HPV type clearance events, with a significant difference for 3 or more HPV type clearance events (HR = 11.46, 95% CI = 6.64 to 19.78). Sensitivity results are described in Table S7. Supplementary Results exploring the association between persistent HPV with abnormal cervical cytology are described in Table S8 and Figure S4.

## Discussion

In this cohort study of young women in Eastern and Southern Africa, HPV clearance events were associated with the greatest risk for HIV seroconversion. Previous studies have linked HPV to HIV acquisition, including a meta-analysis showing a 1.96-fold increased HIV risk after HPV infection.^15^ Our findings suggest that the process of clearing HPV, rather than the presence of a prevalent or persistent infection, may pose an even greater risk of HIV. Specifically, we observed a modest, non-significant 1.34-fold increased HIV risk among women with prevalent 8 cHPV infection. In contrast, HPV clearance was consistently associated with elevated HIV risk, supporting the hypothesis that immune activation during HPV resolution may create conditions favorable for HIV acquisition. This aligns with findings from an observational study involving specimens from South African women in the CAPRISA 004 trial, which suggested that HPV-induced inflammation may recruit HIV target cells to the genital tract, creating an immunological environment conducive to HIV acquisition.^8^^,^^16^^,^^17^

Notably, Liebenberg et al. found greater HIV risk from clearing high-risk HPV types, whereas we observed elevated HIV risk from clearance of both high-risk (8 cHPV, 2vHPV, other cHPV) and low-risk HPV infections, including a non-significant elevated risk for non-oncogenic types HPV 6/11.^8^ This may suggest that the immune activation linked to HPV clearance is not necessarily type-specific, and that clearance of any HPV type could heighten HIV susceptibility. This is further supported by the dose-response relationship observed in our study, where the risk of HIV acquisition increased with the number of HPV types cleared. Given the complex interplay between HPV type, clearance dynamics, and immune response, further research is warranted to clarify the relationship between HPV type and HIV risk, particularly regarding high-risk and low-risk HPV clearance.

A key strength of this study is the monthly collection of endocervical specimens during ASPIRE, offering detailed longitudinal data on HPV and HIV dynamics and an improvement over studies with less frequent sampling.^5^^,^^8^ This study contributes to a deeper understanding of HPV and HIV natural history in a region with a high burden of cervical cancer and HIV prevalence.

Our study has several limitations. First, HPV vaccination status was not collected, potentially confounding associations with HIV and abnormal cytology. However, most participants were likely unvaccinated, as the trial was conducted before national vaccine rollout.^18^^,^^19^ Another limitation is the uncertainty of HIV acquisition timing, as HIV seroconversion occurs after the actual acquisition of the virus. We minimized bias by calculating HIV risk based on HPV results 3 months prior. Lastly, as an observational study, our findings demonstrate correlations rather than causal relationships between HPV and HIV. Future studies with larger sample sizes, advanced methods, like multi-state modeling, and analysis of cytokine concentrations are needed to elucidate the mechanisms and interplay between HPV clearance-associated immune activation and HIV seroconversion.

In conclusion, HIV risk appears linked to generalized inflammation from HPV clearance. These findings underscore the potential role of HPV vaccination not only in preventing HPV-related cancers but also in reducing HIV risk, by preventing HPV acquisition and subsequent clearance events that may drive immune activation. Our findings also support integrating HIV prevention into cervical cancer prevention programs. For example, CIN treatment could be followed by education and provision of HIV prevention methods (such as PrEP) to reduce the risk of HIV. With ongoing efforts to develop therapeutic HPV vaccines, integrating HIV prevention methods alongside HPV treatment could further strengthen prevention strategies and reduce the dual burden of HPV and HIV.

## Supplementary Material

djaf336_Supplementary_Data

## Data Availability

The data that support the findings presented in the study are available upon reasonable request.
