# Supplementary material for: Association of human papillomavirus on risk of HIV acquisition in African women: analyses from MTN-020/ASPIRE
Source: J Natl Cancer Inst. 2025 Nov 19;118(3):485–91. doi: 10.1093/jnci/djaf336 (PMC13017826; doi:10.1093/jnci/djaf336)
Supplement: djaf336_Supplementary_Data [file djaf336_supplementary_data.zip › ASPIRE HIV Pap Appendix_Final_Clean.pdf]

Supplementary Material

Table of Contents

I. Supplementary Methods 2

I.a. Characterizing HPV Infections by HPV Group 2

I.b. Detailed Analysis Description 3

II. References 5

III. Supplementary Tables and Figures 5

III.a. Summary of ASPIRE Specimens Selected for HPV DNA Testing 5

III.b. Supplementary Results 9

III.c. The Association of HPV Persistence with Abnormal Cytology 13

I. Supplementary Methods

I.a. Characterizing HPV Infections by HPV Group

As described in the Methods section, each HPV infection was classified by individual HPV types based on whether they met these five HPV categories: prevalent, persistent, HPV clearance, HPV acquisition, or remaining HPV+. These classifications were then grouped into broader categories based on HPV risk classification (8 cHPV, 2vHPV, other cHPV, lrHPV, HPV6/11) if any HPV type within that group met the criteria at a given time point. **Figure S1** illustrates this approach using bivalent HPV types 16 and 18 for one participant who seroconverted at month 31.

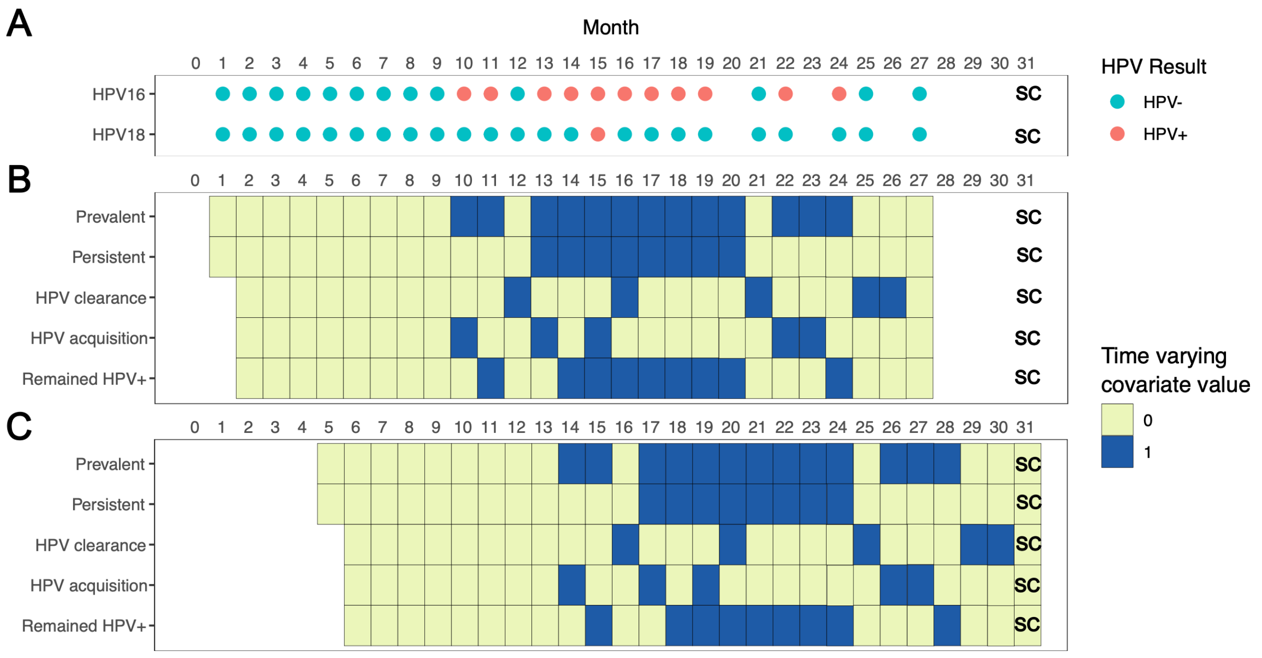

Figure S1. Schematic illustrating a single participant's (A) HPV results for HPV16/18, (B) corresponding time-varying covariate values, including prevalence, persistence, HPV clearance, HPV acquisition, and remaining HPV+, and (C) the time-varying covariate values shifted three months forward used in time-varying covariate Cox models to reflect the risk of HIV seroconversion based on HPV status three months prior. A time-varying covariate value was assigned a value of "1" if any HPV result within the bivalent HPV grouping met the criteria for that covariate at the time point. SC denotes the seroconversion visit. Missing HPV data was imputed with last observation carried forward. HPV characterizations for HPV clearance, HPV acquisition, and remaining HPV+ were not assigned at the first swab, as these classifications require two swabs for determination.

Figure S1 represents a participant who seroconverted. As shown in panel C, in the time-varying Cox model, the start time was set to month 5 for prevalent and persistent HPV classifications, and to month 6 for HPV clearance, acquisition, and remaining HPV positive. The event time was the time from month 5 to month 31 (the month of seroconversion). Participants who did not seroconvert or whose last HPV result was more than three months prior to the seroconversion time point were censored three months after their last HPV result.

I.b. Detailed Analysis Description

Data Cleaning for Demographics:

Demographics were summarized for each visit month that a participant had in the ASPIRE study.

Age was summarized by age at enrollment into three groups: ages (17,21], (21,26], and (26,45]. This was applied to all months that a participant was enrolled in ASPIRE.

Education was summarized into whether secondary education was completed. Secondary education not completed included any response of "no schooling", "primary school, complete", "primary school, not complete", and "secondary school, not complete". All other responses were grouped into secondary school completed. This was applied to all ASPIRE visit months for that participant.

Condom use at last sex encounter was summarized into a binary variable of 0/1. 1 was classified as a "female condom", "male condom", or "both" responses. Any missing data was imputed with last observation carried forward. If the first observation was missing, then the next observation was carried backward.

Having multiple sex partners was summarized into a binary variable of 0/1. 0 was assigned if (1) the participant said they had a primary sex partner (PSP) and 0 other partners other than the PSP, (2) the participant said they did not have a PSP nor other partners other than a PSP, and (3) the participant said they did not have a PSP but had 1 sex partner other than a PSP. A value of 1 was assigned for this column if the participant said they had a PSP and one or more partners other than their PSP, or if the participant said they did not have a PSP and two or more partners other than a PSP. Any missing data was imputed with last observation carried forward. If the first observation was missing, then the next observation was carried backward.

Syphilis, trichomoniasis, gonorrhea, and chlamydia were each summarized as a 1/0 binary variable. For syphilis, a value of "1" was assigned to a reactive test and a positive confirmatory test, while a value of "0" was assigned to a reactive test and negative confirmatory test or non-reactive test. Any missing data for each of these STIs was imputed with the last observation carried forward. If the first observation was missing, then the next observation was carried backward. All these STIs were then summarized into an overall STI binary grouping. "1" was assigned if the participant was positive for any of the four tested STIs, and "0" was assigned if the participant was negative for all tested STIs during each visit.

Weighting:

The 1,215 participants who had their specimens tested for HPV DNA (in addition, consented for their specimens to be used for future research and were part of the 2,614 participants included in the original ASPIRE analysis), were weighted back to the 2,359 ASPIRE participants who consented for their specimens to be used for future research.

For participants who seroconverted during ASPIRE or had an abnormal pap smear, weighting was done by HIV status and pap smear result. This is because when selecting which specimens to test for HPV DNA, we over-sampled for participants who seroconverted or who had an abnormal pap smear. For participants who were HIV- and had a normal pap smear, weighting was done by study site and age group (17-21, 21-26, 26-

45). Weights were calculated as the count of participants in each subgroup from the full ASPIRE study divided by the count of participants in each subgroup whose specimens were tested for HPV DNA.

Classifying HPV Results:

We classified HPV infections as prevalent (HPV+), persistent (HPV+ for the same HPV type over a span of at least 4 months, as defined in previous studies), HPV clearance (HPV- with a previous swab that was HPV+ for the same type), HPV acquisition (HPV+ with a previous swab that was HPV- for the same type), and remaining HPV positive (HPV+ with a previous swab that was HPV+ for the same type).

HPV infections were classified into the eight carcinogenic HPV types relevant for screening per the WHO (8 cHPV – HPV 16/18/31/33/35/45/52/58), bivalent vaccine (2vHPV – HPV 16/18), other carcinogenic HPV types (other cHPV – HPV 39/51/56/59/66/68/73), low-risk HPV types (lrHPV – HPV 26/40/42/53/54/61/69/70/82), non-carcinogenic HPV types (HPV 6/11), and any HPV type [1]. High-risk and low-risk classifications followed the International Agency for Research on Cancer classification scheme [1, 2].

Time Varying Covariate Cox Models:

We first filtered for participants who had 3 or more specimens tested for HPV DNA, were part of the original 2,614 participants from the original ASPIRE analysis, and consented to have their specimens used for future research.

We summarized the HPV results based on the HPV type grouping (8 cHPV, 2vHPV, other cHPV, lrHPV, and HPV 6/11). Separate columns were created which summarized for each unique participant and visit using a binary 0/1 if there was any HPV type in the grouping that was prevalent, persistent, HPV clearance, HPV acquisition, or remaining HPV+ (5 summary columns).

A new column was created that indicated which study month would be 3 months in the future (3+current study month). This is because we were ultimately looking at the risk of HIV acquisition based on the HPV results 3 months prior.

A separate dataframe was then created that uses the first and last ASPIRE visit month for each participant to create a table with all the incremental months within those month ranges. For example, if a participant entered the study at month 0 and left at month 12, they would have 13 rows in this dataframe for each month they were in the study.

Using this new dataframe and using the *tmerge* (from the "survival" library in R) function, for each participant, the event time was set as the month of seroconversion (for participants who seroconverted) or the last visit during the ASPIRE trial (for participants who did not seroconvert). Columns were then merged in this order: age group, sample weight, pap result, study site, marriage status, secondary education completed, condom used at last sex encounter, whether or not they have multiple sex partners, STIs, study arm, and the HPV summary results of prevalent, persistent, HPV clearance, HPV acquisition, and remaining HPV+. Key to joining the HPV summary results was that we joined the observed visit month by the visit month 3 months in the future for the HPV results.

A final dataframe was created with all the demographics, event status (based on seroconversion visit), and HPV results (3 months prior) for all participants who had at least 3 specimens tested for HPV DNA, for the entire duration that they were enrolled in the ASPIRE study.

However, we did not have HPV DNA results for every time point that these participants were in the ASPIRE trial. We want the start time to be the month of their second HPV DNA result plus 3 months. We needed their second HPV DNA result because that is the first timepoint when we can make a classification of HPV clearance, acquisition, etc., since these HPV characterizations need 2 HPV results. The plus 3 is because each HPV DNA result is assigned to the study visit 3 months in the future. Additionally, for the participants who did not seroconvert, we censored them at the time of their last HPV DNA result plus 3. This way the censored participants were treated in the same way as participants with an HIV endpoint: participants with an endpoint had their last observation at their seroconversion month (which for most participants is 4 months after their last HPV DNA result), and participants who were censored had their last observation 4 months after their last HPV DNA result. Note that any participant who seroconverted but whose last HPV result was more than 4 months from seroconversion was also censored.

After this, the dataframe was finally used to run the Cox models. The following chunk of code shows how the Cox models were run. The highlighted variable in yellow was adjusted for each separate analysis (HPV clearance, HPV acquisition, remaining HPV+, etc.)

coxph_tradDef <- coxph(Surv(tstart, tstop, event) ~ age_grp + strata(site) + secondEduComplete + lvsc_bin + multPart + any_sti + arm + clear, data = tmerge_tradDef, weights = sample_weight)

II. References

1. *Cervical Cancer Screening: IARC Handbooks of Cancer Prevention, Volume 18*. Lyon: International Agency for Research on Cancer (IARC); 2022.
2. Bouvard V, Baan R, Straif K, *et al.* A review of human carcinogens--Part B: biological agents. Lancet Oncol 2009;10(4):321-2.

III. Supplementary Tables and Figures

III.a. Summary of ASPIRE Specimens Selected for HPV DNA Testing

Figure S2. Count of endocervical swabs collected at each visit month during the ASPIRE study compared to the swabs tested for HPV DNA.

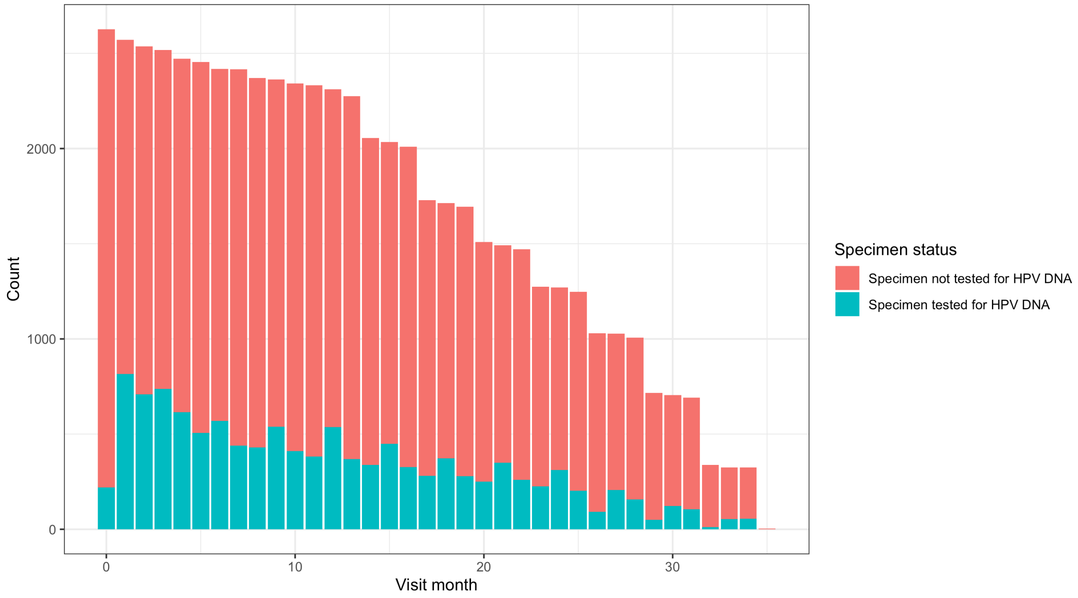

Figure S3. Out of the 1,215 women with at least one swab tested for HPV DNA during the ASPIRE study, the count of swabs tested for HPV DNA at each visit month.

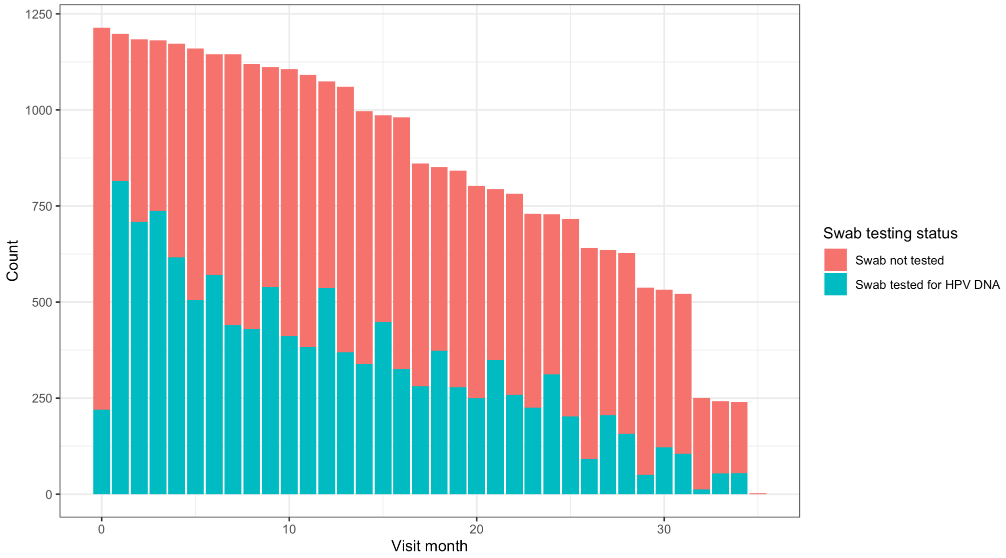

Table S1. Count of participants with missing swabs (defined as swab collected during the ASPIRE trial but not tested for HPV DNA).

Number of missing swabs (swab collected during ASPIRE but not tested for HPV DNA)	Count of participants	Cumulative percent of participants (N=2,359)
0	57	2.42%
1	49	4.49%
2	34	5.93%
3	47	7.93%
4	72	10.98%
5	76	14.20%
6	72	17.25%
7	79	20.60%
8	130	26.11%
9	46	28.06%
10	31	29.38%
11	20	30.22%
12	36	31.75%
13	19	32.56%
14	121	37.69%
15	28	38.87%
16	22	39.81%
17	108	44.38%
18	30	45.65%
19	19	46.46%
20	165	53.45%
21	30	54.73%
22	18	55.49%
23	151	61.89%
24	23	62.87%
25	17	63.59%
26	145	69.73%
27	5	69.94%
28	13	70.50%
29	269	81.90%
30	81	85.33%
31	119	90.38%
32	109	95%
33	36	96.52%
34	16	97.20%
35	65	99.96%

Number of missing swabs (swab collected during ASPIRE but not tested for HPV DNA)	Count of participants	Cumulative percent of participants (N=2,359)
36	1	100%

Table S2. Out of the 1,215 participants with at least one swab tested for HPV DNA, the number of missing HPV DNA results between the first and last swab.

Number of missing HPV DNA tests within the first and last swabs tested for HPV DNA	Count of participants	Cumulative percent of participants (N=1,215)
0	417	34.32%
1	67	39.84%
2	73	45.84%
3	84	52.76%
4	68	58.35%
5	29	60.74%
6	52	65.02%
7	58	69.79%
8	56	74.40%
9	16	75.72%
10	21	77.45%
11	12	78.44%
12	14	79.59%
13	9	80.33%
14	18	81.81%
15	10	82.63%
16	15	83.87%
17	7	84.44%
18	15	85.68%
19	8	86.34%
20	17	87.74%
21	6	88.23%
22	5	88.64%
Only 1 swab tested for HPV DNA	138	100%

Table S3. The number of swabs tested for HPV DNA out of the 1,215 participants with HPV DNA results.

Number of swabs tested for HPV DNA	Count of participants	Cumulative percent of participants (N=1,215)
1	138	11.36%
2	127	21.81%
3	161	35.06%
4	103	43.54%
5	81	50.21%

Number of swabs tested for HPV DNA	Count of participants	Cumulative percent of participants (N=1,215)
6	30	52.67%
7	27	54.98%
8	34	57.70%
9	47	61.56%
10	43	65.19%
11	34	67.90%
12	13	68.97%
13	29	71.36%
14	44	74.98%
15	15	76.21%
16	14	77.37%
17	22	79.18%
18	16	80.49%
19	12	81.48%
20	14	82.63%
21	20	84.28%
22	9	85.02%
23	18	86.50%
24	27	88.72%
25	40	92.02%
26	18	93.50%
27	26	95.64%
28	14	96.79%
29	12	97.78%
30	2	97.94%
31	15	99.18%
32	2	99.34%
33	1	99.42%
34	7	100%

III.b. Supplementary Results

Table S4. Demographic characteristics and sexual behaviors of ASPIRE participants who were not included in the observational study.

	Participants who did not seroconvert (N=1,358)	Participants who seroconverted (N=60)	Total (N=1,418)
Characteristics at enrollment			
Mean age (SD)	27.5 (6.2)	25.6 (5.5)	27.4 (6.2)
Education completed			
No schooling completed	130 (10%)	6 (10%)	136 (10%)
Primary school	578 (43%)	28 (48%)	606 (43%)
Secondary school	563 (41%)	21 (36%)	584 (41%)
College or university	89 (7%)	3 (5%)	92 (7%)
Country			
Malawi	108 (8%)	2 (3%)	110 (8%)
South Africa	621 (46%)	47 (81%)	668 (47%)
Uganda	184 (14%)	3 (5%)	187 (13%)
Zimbabwe	447 (33%)	6 (10%)	453 (32%)
Study arm			
Dapivirine	689 (51%)	26 (45%)	715 (50%)
Placebo	671 (49%)	32 (55%)	703 (50%)
Married			
Yes	633 (47%)	4 (7%)	637 (45%)
No	727 (53%)	54 (93%)	781 (55%)
Missing			
Time varying characteristics [a]			
Condom use during last sex encounter			
Yes	1100 (81%)	38 (68%)	1138 (80%)
No	262 (19%)	18 (32%)	280 (20%)
Multiple sex partners			
Yes	406 (30%)	20 (33%)	426 (30%)
No	952 (70%)	40 (67%)	992 (70%)
Any STIs [b]			

Yes	550 (41%)	27 (45%)	577 (41%)
No	808 (59%)	33 (55%)	841 (59%)

[a] Summarized as having the characteristic at any time during the ASPIRE trial before seroconversion (for participants who seroconverted) or before the product use end visit (participants who did not seroconvert).

[b] Syphilis, trichomoniasis, gonorrhea, and chlamydia.

Table S5. HPV prevalence at the baseline swab[a] for all participants tested for HPV DNA in the observational study cohort.

HPV group	All participants tested for HPV DNA	HIV cohort, censored participants	HIV cohort, endpoint participants
2v types	0.178 (216/1215)	0.176 (150/850)	0.253 (23/91)
8 cHPV	0.449 (546/1215)	0.447 (380/850)	0.626 (57/91)
Other cHPV	0.332 (403/1215)	0.333 (283/850)	0.462 (42/91)
lrHPV	0.354 (430/1215)	0.356 (303/850)	0.429 (39/91)
HPV 6/11	0.085 (103/1215)	0.087 (74/850)	0.132 (12/91)
Any HPV type	0.707 (859/1215)	0.709 (603/850)	0.813 (74/91)

2v, bivalent vaccine type; 8 cHPV, eight carcinogenic HPV types relevant for screening per the WHO; other cHPV, other carcinogenic HPV types; lrHPV, low-risk HPV types.

[a] Baseline swab is defined as the first swab tested for HPV DNA.

Table S6. Incidence of persistent HPV for the high-risk nonavalent vaccine HPV types. Persistence is defined as at least 4.5 months of positive HPV results for any single HPV type.

HPV types 16/18/31/33/45/52/58					
**N women from ASPIRE with HPV test results**	**N women HPV 16/18/31/33/45/52/58 naive at baseline**	**N women with follow up time > 4.5 months**	**Incident persistent HPV 16/18/31/33/45/52/58**	**Woman-yr of follow up**	**Incidence of persistent HPV 16/18/31/33/45/52/58 per 100 woman-yr**
1215	718	403	77	584	13.18
HPV types 31/33/45/52/58					
**N women from ASPIRE with HPV test results**	**N women HPV 31/33/45/52/58 naive at baseline**	**N women with follow up time > 4.5 months**	**Incident persistent HPV 31/33/45/52/58**	**Woman-yr of follow up**	**Incidence of persistent HPV 31/33/45/52/58 per 100 woman yr**
1215	855	490	73	743.83	9.81

Table S7. Sensitivity analysis of adjusted hazard ratios from Cox time-varying models of HIV risk based on time-varying HPV status three months prior. HPV clearance for this sensitivity analysis is defined as a negative HPV result following a persistent infection – characterized by at least 4 months of consecutive HPV-positive results for a specific HPV type. The hazard ratios derived from this definition are generally higher than those from the main analysis that defines HPV clearance as any negative HPV result following a positive one, reinforcing the association between HPV clearance and an increased risk of HIV acquisition.

HPV Characterization		Person -Years	Events	Incidence Rate (per 1,000 person-years)	Hazard Ratio (95% CI)	P-value
All HPV types	Ref. No HPV clearance	943.7	65	68.9		
	HPV clearance	78.2	26	332.6	4.27 (2.67-6.83)	<0.001
8 cHPV	Ref. No HPV clearance	992.2	79	79.6		
	HPV clearance	29.7	12	404.5	4.09 (2.21-7.56)	<0.001
2v types	Ref. No HPV clearance	1010.5	88	87.1		
	HPV clearance	11.3	3	264.7	2.04 (0.67-6.21)	0.254
Other cHPV	Ref. No HPV clearance	999.1	87	87.1		
	HPV clearance	22.8	4	175.8	1.50 (0.55-4.13)	0.462
lrHPV	Ref. No HPV clearance	996.2	85	85.3		
	HPV clearance	25.6	6	234.5	2.10 (0.94-4.72)	0.107
HPV 6/11	Ref. No HPV clearance	1018.0	89	87.4		
	HPV clearance	3.8	2	521.7	4.75 (1.18-19.16)	0.054

III.c. The Association of HPV Persistence with Abnormal Cytology

Cervical cytology smears were collected at the Product Use End Visit for consenting participants. We wanted to contribute to the literature characterizing the association of HPV infection on abnormal cervical cytology results.

Participants with a cervical cytology smear and HPV-DNA tested endocervical swabs were included in the analysis if they had at least three HPV-DNA results, with at least one result within 12 months of the cytology smear. The purpose of this was to evaluate clearance of persistent infection, requiring at least three HPV-DNA results.

Prevalent infections were defined by a single HPV+ result, persistent infections defined by consecutive HPV+ results at least four months apart, and clearance of persistent infections defined by a single HPV- result following a persistent infection. Cytology smears were reported as normal or abnormal (ASCUS, ASC-H, LSIL, and HSIL).

Baseline characteristics collected at the enrollment visit (age, education, country, study arm, and marital status) and time-varying characteristics (condom use, multiple partners, and STIs) collected at follow-up visits were compared using Chi-square tests for categorical variables and analysis of variance tests for continuous variables.

Logistic regression models estimated associations between persistent HPV infection of any type in the year before the cervical cytology smear and an abnormal cytology result. Additional analyses were performed for 2vHPV, 9vHPV, non-vaccine hrHPV, and non-vaccine lrHPV. These HPV groups were analyzed because prophylactic vaccines have high efficacy in preventing persistent infection, making them modifiable risk factors for abnormal cytology outcomes. Analyses also assessed whether the number of unique persistent HPV types was associated with abnormal cytology. Finally, we evaluated the association of persistent infection and clearance of persistent infection on abnormal cytology. These exposures were analyzed separately to assess their independent associations with abnormal cytology, and were adjusted by age group at enrollment (ages 18-21, 22-26, and 27+), study site, completion of secondary education, condom use at the last sex encounter, multiple sex partners, the presence of any STIs, and study arm.

A total of 11,778 specimens from 1,215 ASPIRE participants were tested for HPV-DNA, including 126 participants who had an abnormal cytology and 1,005 participants with a normal cytology result. Based on the inclusion criteria for this analysis, 77 participants included in the analysis had an abnormal cervical cytology while 557 participants had a normal cervical cytology.

Significant demographic differences were observed between cohorts by age and country. Participants with an abnormal cervical cytology were younger than those with normal results (mean age of 23.6 vs. 27.1, P<0.001). Most cytology results were from South Africa (80%), followed by Zimbabwe (13%), Malawi (4%), and Uganda (3%). Abnormal cytology rates varied significantly by country (P<0.001): none were reported in Uganda or Zimbabwe, while 43% of results from Malawi and 13% from South Africa were abnormal. No significant differences were found by education, study arm, marital status, condom use, multiple sex partners, or STIs (**Table S8**).

Table S8. Demographic characteristics and sexual behaviors of HIV and Pap study participants at enrollment and follow-up.

	Normal cervical cytology (N=557)	Abnormal cervical cytology (N=77)	Total (N=634)	P-value[a]
Characteristics at enrollment				
Mean age (SD)	27.1 (6)	23.6 (4.4)	26.7 (6)	<0.001
Education completed				0.058
No schooling completed	26 (5%)	7 (9%)	33 (5%)	
Primary school	261 (47%)	26 (34%)	287 (45%)	
Secondary school	237 (43%)	36 (47%)	273 (43%)	
College or university	33 (6%)	8 (10%)	41 (7%)	
Country				<0.001

Malawi	16 (3%)	12 (16%)	28 (4%)	
South Africa	441 (79%)	65 (84%)	506 (80%)	
Uganda	19 (3%)	0 (0%)	19 (3%)	
Zimbabwe	81 (15%)	0 (0%)	81 (13%)	
Study arm				0.808
Dapivirine	280 (50%)	37 (48%)	317 (50%)	
Placebo	277 (50%)	40 (52%)	317 (50%)	
Married				0.080
Yes	125 (22%)	10 (13%)	135 (21%)	
No	432 (78%)	67 (87%)	499 (79%)	
Time varying characteristics [b]				
Condom use during last sex encounter				0.617
Yes	453 (81%)	65 (84%)	518 (82%)	
No	104 (19%)	12 (16%)	116 (18%)	
Multiple sex partners				0.809
Yes	70 (13%)	11 (14%)	81 (13%)	
No	487 (87%)	66 (86%)	553 (87%)	
Any STIs [c]				0.499
Yes	163 (29%)	26 (34%)	189 (30%)	
No	394 (71%)	51 (66%)	445 (70%)	

SD, standard deviation; STI, sexually transmitted infection

[a] Estimated from analysis of variance tests for continuous variables and Chi-square tests for categorical variables.

[b] Summarized as having the characteristic at any time before seroconversion (for seroconvertors in the HIV study), before the product use end visit (for non-seroconverters in the HIV study), or at any time within one year of the cervical cytology (for participants in the Pap Study).

[c] Syphilis, trichomoniasis, gonorrhea, and chlamydia.

Persistent HPV infection within 12 months of a cervical cytology smear was associated with a 5.40 (2.11-13.81) times greater odds of abnormal cytology, as shown in **Figure S4**. Clearing a persistent infection also raised the odds (OR 4.52, 95% CI: 2.36-8.66), while prevalent infections did not. Among HPV groupings, persistent 9vHPV infections had the greatest odds of abnormal cytology (7.84, 95% CI: 4.09-15.05), followed by non-vaccine hrHPV (OR 3.60, 95% CI: 1.81-7.14), 2vHPV (OR 2.42, 95% CI: 1.35-4.35), and non-vaccine lrHPV (OR 2.18, 95% CI: 1.24-3.84).

Figure S4. Adjusted odds ratios (aOR) for the association between the number of persistent HPV types over 12 months and an abnormal cytology. Logistic regression models were adjusted by study site, age at enrollment, completion of secondary education, condom use at the last sex encounter, having multiple sex partners, the presence of any STIs, and ASPIRE randomization group. Footnotes: [a]Reference: no prevalent infection; [b]Reference: no persistent infection; [c]Reference: no clearance of persistent infection; [d]Reference: zero persistent HPV types.

HPV Characterization		Number of participants with the HPV event at any time: Abnormal cytology (N=77)	Normal cytology (N=557)	Odds Ratio (95% CI)	P-value
Any HPV	Prevalent [a]	75	501	2.86 (0.65-12.66)	0.165
	Persistent [b]	73	400	5.40 (2.11-13.81)	<0.001
	Clearance of persistent infection [c]	55	192	4.52 (2.36-8.66)	<0.001
2vHPV	Persistent [a]	33	105	2.42 (1.35-4.35)	0.003
9vHPV	Persistent [a]	67	252	7.84 (4.09-15.05)	<0.001
Non-vaccine HR	Persistent [a]	63	268	3.60 (1.81-7.14)	<0.001
Non-vaccine LR	Persistent [a]	46	209	2.18 (1.24-3.84)	0.007
Any HPV	1 persistent HPV type [d]	3	136	0.56 (0.10-3.02)	0.498
	2 persistent HPV types [d]	10	95	4.07 (1.34-12.33)	0.013
	3+ persistent HPV types [d]	60	167	11.89 (4.31-32.80)	<0.001
	Number of persistent HPV types (continuous)			1.42 (1.27-1.59)	<0.001
9vHPV	1 persistent HPV type [d]	5	148	1.08 (0.27-4.35)	0.916
	2 persistent HPV types [d]	23	91	10.31 (3.74-28.45)	<0.001
	3+ persistent HPV types [d]	45	108	21.21 (7.38-60.97)	<0.001
	Number of persistent HPV types (continuous)			1.70 (1.44-2.02)	<0.001

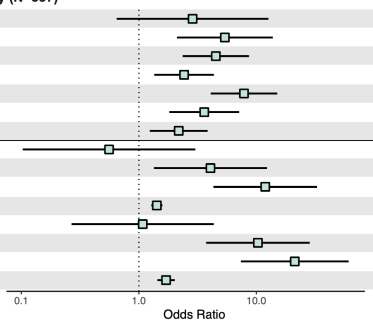

A dose-response relationship was also observed: for 9vHPV, odds of abnormal cytology increased to 10.31 (95% CI: 3.74-28.45) with two persistent HPV types, and 21.21 (95% CI: 7.38-60.97) with three or more persistent HPV types, when compared with zero persistent HPV types. A similar trend was noted for any HPV type (OR: 4.07, 95% CI: 1.34-12.33 for two types, OR: 11.89, 95% CI: 4.31-32.80 for ≥3 types).

Our finding that persistent HPV infection, especially with 9vHPV types, is strongly associated with abnormal cytology aligns with previous studies showing that persistent HPV is linked to an increased risk of ASCUS, CIN1/LSIL, and CIN2-3/HSIL+. These results underscore the 9vHPV vaccine's key role in preventing persistent infections most likely to persist and progress to cervical neoplasia. This analysis also contributes to a deeper understanding of HPV's natural history in the region with the highest burden of cervical cancer and HIV prevalence.
